# Fibroblasts from Niemann Pick type C patients are more prone to autophagy induction than fibroblasts from healthy individuals under starvation conditions and Chloroquine treatment

**DOI:** 10.1002/alz70855_101377

**Published:** 2025-12-23

**Authors:** Luis‐Jesus Miliar‐Martinez, Rebeca‐Leticia Alcala‐Flores, Maria‐del‐Carmen Silva‐Lucero, Maria‐del‐Carmen Cardenas‐Aguayo

**Affiliations:** ^1^ UNAM, School of Medicine, CDMX, DF, Mexico; ^2^ Biochemistry, Master in Science Program, UNAM, CDMX, DF, Mexico; ^3^ UNAM, School of Medicine, Department of Physiology, CDMX, DF, Mexico

## Abstract

**Background:**

Niemann‐Pick disease type C (NPC) is a neuro‐visceral pathology caused by autosomal recessive mutations in *NPC1* and *NPC2* genes resulting in an endolysosomal accumulation of sphingomyelin and non‐esterified cholesterol mainly in cells of the brain, liver, spleen and immune system (Berry‐Kravis, 2020). Autophagy represents the main lysosomal pathway by which cells degrade damaged constituents and get protection by eliminating toxic organelles and peptides, resulting in apoptosis inhibition (Nixon, 2024). In this way, the deficiency in lipid degradation in lysosomal disorders correlates with an alteration in autophagic flux.

**Method:**

Induction of autophagy was performed by serum starvation for 6 h in cultures of 3 fibroblast cell lines from apparently healthy individuals and 3 fibroblast cell lines from NPC patients to determine if there were differences in the autophagic process between controls and patients. We also tested the effect of Chloroquine 5 mM for 6 h on these cultured cells. Autophagy induction was confirmed using the Cyto‐ID autophagy detection kit (ENZO). We analyzed the expression of autophagic pathway proteins: LC3‐II and p62 by Western Blotting. We analyzed the transcriptome of both control and patient cell lines using differential expression analysis to identify the mechanisms involved in the autophagic pathway.

**Result:**

Treatment with Chloroquine had a more significant effect on the induction of autophagy in cells from patients with NPC as compared to control cultures; an increase in the presence of cytoplasmic autophagy vacuoles, a greater expression of LC3‐II and a reduction in the expression of p62 in fibroblasts from NPCs patients was confirmed. Six hours of serum starvation did not show a significant difference in autophagy markers compared to the controls.

**Conclusion:**

NPC fibroblast cells were more prone to induction of the first phases of autophagy when treated with Chloroquine under starvation conditions compared to fibroblasts from healthy individuals. This result was confirmed by Cyto‐ID autophagy assay and LC3‐II increase expression. These cells derived from NPC patients constitute an optimal model to study this neurodegenerative disease *in vitro*.

